# Successful prehospital double sequential external defibrillation in a 15-year-old with hypertrophic cardiomyopathy

**DOI:** 10.1016/j.resplu.2026.101368

**Published:** 2026-05-21

**Authors:** F. Weilbacher, D. Matthé, J. Fricke, E. Amr, M. Dietrich, E. Popp, S. Katzenschlager

**Affiliations:** a Heidelberg University, Medical Faculty Heidelberg, Department of Anaesthesiology, Heidelberg, Germany; b Heidelberg University, Medical Faculty Heidelberg, Department of Cardiology, Heidelberg, Germany

**Keywords:** Cardiopulmonary resuscitation, Electric countershock, Adolescent, Child, Ventricular fibrillation

## Abstract

We present the case of a 15-year-old boy with hypertrophic cardiomyopathy who suffered out-of-hospital cardiac arrest. After failure of conventional defibrillation to terminate refractory ventricular fibrillation, double sequential external defibrillation was used successfully, resulting in return of spontaneous circulation. Ultimately, the patient survived neurologically intact.

**Background:**

Hypertrophic cardiomyopathy is the most common monogenic cardiac disorder. Paediatric hypertrophic cardiomyopathy patients have a higher risk of ventricular fibrillation and sudden cardiac death compared to adults. Double sequential external defibrillation has been successfully used for refractory ventricular fibrillation in the adult population, but is not currently mentioned in paediatric life support guidelines.

**Case presentation:**

We present the case of a 15-year-old boy with hypertrophic cardiomyopathy who suffered out-of-hospital cardiac arrest and persistent ventricular fibrillation. After three failed shocks with an anterolateral defibrillation pad position, double sequential external defibrillation was successfully used as a rescue therapy. A bundle of advanced prehospital interventions, including cerebral near-infrared spectroscopy, invasive blood pressure monitoring and transoesophageal echocardiography, was used to stabilise the patient who ultimately survived without neurological impairment.

**Discussion:**

This case demonstrates the potential benefit of a double sequential external defibrillation strategy in paediatric patients with refractory ventricular fibrillation. Adequate training is necessary to optimise the efficacy of double sequential external defibrillation. Bundles of advanced prehospital interventions could further contribute to favourable outcomes after prolonged paediatric resuscitation. Registries should be used to systematically capture potential paediatric double sequential external defibrillation candidates to facilitate high-quality observational studies.

## Background

Hypertrophic cardiomyopathy (HCM) is the most common monogenic cardiac disorder and carries a substantial risk of ventricular fibrillation (VF) and sudden cardiac death. Notably, paediatric HCM patients have a higher risk of VF and sudden cardiac death compared to adults.^1^, ^2^

Overall, shockable rhythms are infrequent in children, with the highest rate among those aged 13–17 in Germany, at 21%.^3^ Of those needing defibrillation, one-third received more than three shocks.^3^ In the adult population, double sequential external defibrillation (DSED) can be used for refractory ventricular fibrillation (VF).^4^ This treatment option is currently not mentioned in the paediatric life support (PLS) guidelines.^5^

## Case presentation

We present the case of a 15-year-old boy with out-of-hospital cardiac arrest (OHCA) and persistent VF. The patient had previously been diagnosed with hypertrophic cardiomyopathy (HCM) and was under regular cardiological supervision. Due to a stable course of the disease, he had been cleared for moderate exercise. During basketball training in a public sports hall, the patient suffered a bystander-witnessed collapse. A physician who was training on a nearby court was called to the scene, confirmed cardiac arrest and initiated chest compressions with a presumed maximum no-flow time of 5 min.

An ambulance staffed with paramedics, a physician response car, and a Medical Intervention Car was dispatched to the patient. The Medical Intervention Car is a physician-staffed unit specialised in treating critically ill patients and capable of providing advanced interventions in the field.

**Fig. 1:**
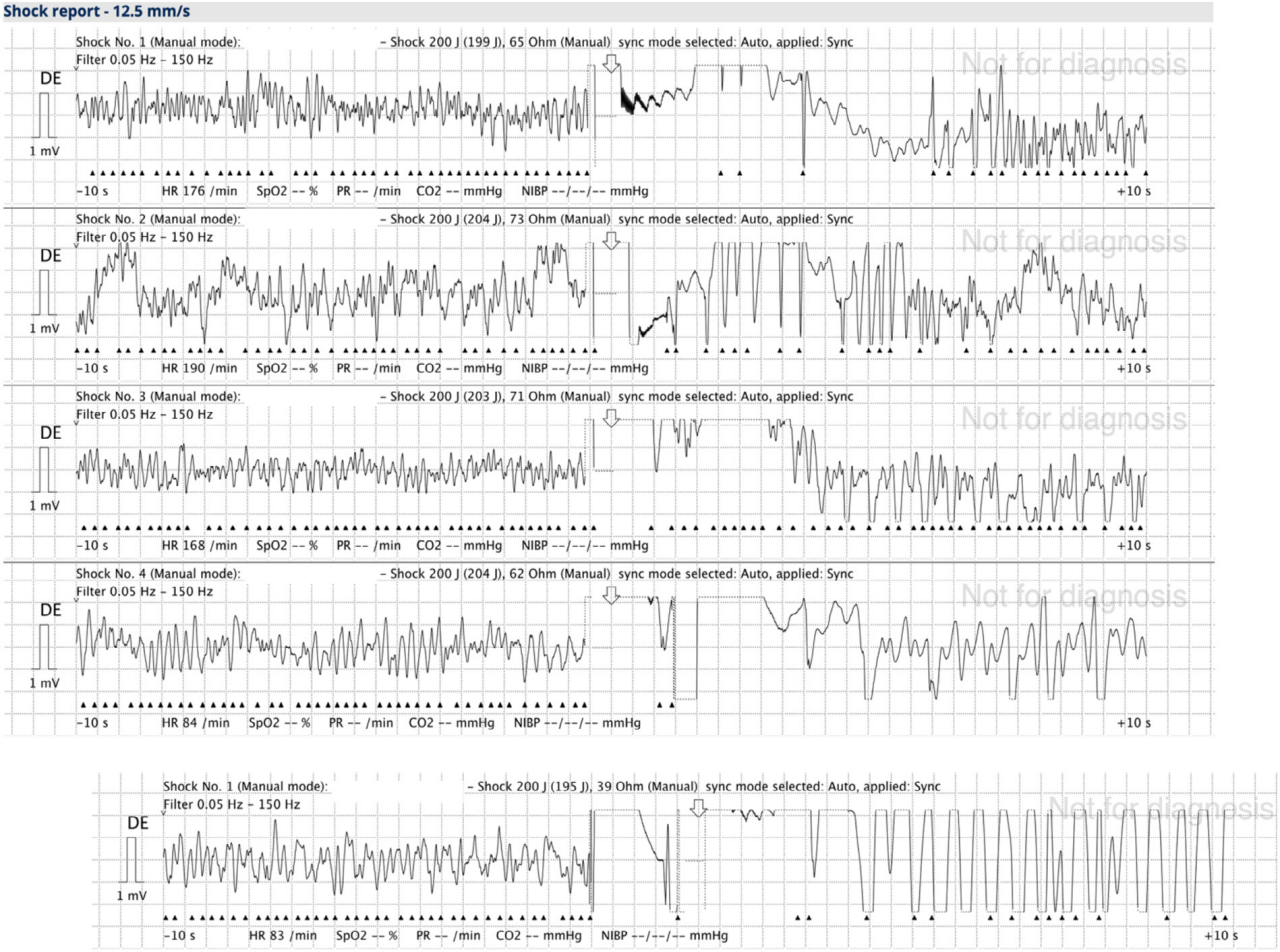
**Shock report. The first three shocks were delivered in an anterolateral position by a single device. Double sequential external defibrillation was performed according to the trial by Cheskes et al.**^3^

Initial advanced life support (ALS) was provided by the teams of the ambulance and the physician response car, who arrived on scene 8 min after the emergency call. Despite the patient being an adolescent, the team decided to follow adult recommendations with regard to chest compressions, drug dosing and defibrillation strategy due to an estimated bodyweight of 85 kg. Initially, the presenting rhythm of VF could be terminated with a single biphasic shock of 200 J using an anterolateral pad position (corpuls3T with corPatch Easy adult size defibrillation electrodes, GS Elektromedizinische Geräte G. Stemple GmbH, Kaufering, Germany). However, VF recurred within seconds after the first shock. After three failed shocks with identical, maximum energy levels and pad position, as well as administration of the initial doses of amiodarone (300 mg) and adrenaline (1 mg), the team decided to use DSED as a rescue therapy (Fig. 1).

A second defibrillator (corpuls3T with corPatch Easy adult size defibrillation electrodes, GS Elektromedizinische Geräte G. Stemple GmbH, Kaufering, Germany) was used for this purpose with the pads in the anteroposterior position, and both defibrillators were discharged in rapid succession. Automatic T-wave synchronisation was not deactivated on either defibrillator. At the subsequent rhythm check performed two minutes after DSED, a palpable carotid pulse was detected, and return of spontaneous circulation (ROSC) was declared after a total of 8 min of ALS, including three unsuccessful conventional defibrillation attempts and one DSED. The Medical Intervention Car team arrived shortly after the first ROSC. Pre-shock pauses were 4, 5, 7, and 12 s, with a median of 6.0 s [IQR 4.75–8.25]. Post-shock pauses were 1, 1, 1, and 2 s, with a median of 1.0 s [IQR 1.00–1.25].

**Fig. 2:**
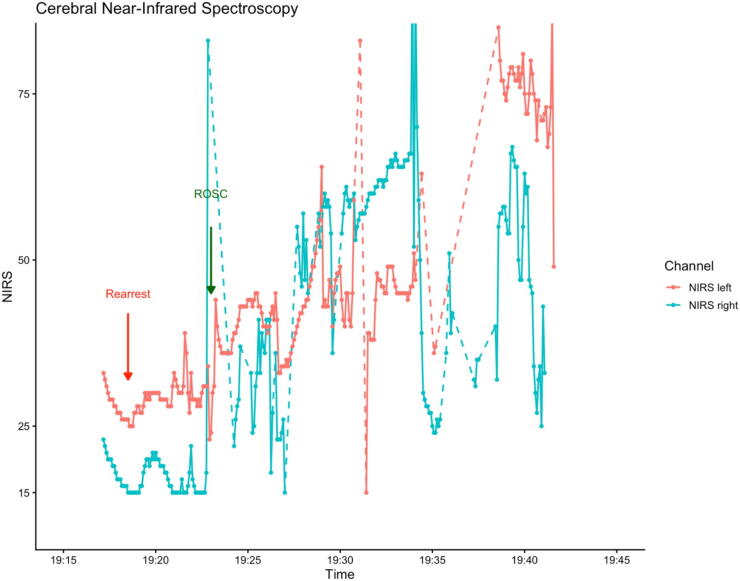
**Cerebral near-infrared spectroscopy. Dashed lines indicate links between the two most recent recorded values. Solid lines represent continuous values**.

Transoesophageal echocardiography (TOE) and cerebral near-infrared spectroscopy (cNIRS) were applied. The TOE showed hypertrophic myocardium and globally severely impaired contractility. Right ventricular dilatation or pericardial effusion could be ruled out. Initial cNIRS values were below the physiological range (left: 33%, right: 23%), indicating poor cerebral tissue oxygenation (Fig. 2).

In line with these findings, the patient lost cardiac output again shortly after, despite the use of vasoactive drugs (cafedrine/theodrenaline, noradrenaline) and the presence of a potentially perfusing cardiac rhythm (pulseless electrical activity).

Chest compressions were restarted immediately, and an additional dose of adrenaline was administered based on the TOE finding of decreased contractility.

In parallel, 4-French femoral arterial and venous sheaths were placed to prepare for potential extracorporeal cardiopulmonary resuscitation (eCPR) and to enable invasive blood pressure monitoring and arterial blood gas analysis.

Sustained ROSC was achieved after another 4 min of CPR, so eCPR remained on standby but ultimately was not required.

Blood gas analysis was obtained after the second ROSC and revealed profound metabolic and respiratory acidosis (Table 1); therefore, ventilation was adjusted accordingly, and 100 mL of 8.4% sodium bicarbonate was administered.

**Table 1:** Arterial blood gas analysis after ROSC.

**Parameter**	**Value**
pH	6.9
pCO_2_ [mmHg]	95
pO_2_ [mmHg]	194
HCO_3_^−^ [mmol/L]	18.2
BE [mmol/L]	−15
Lactate [mg/dL]	98
Glucose [mg/dL]	227
Sodium [mmol/L]	141
Potassium [mmol/L]	3.9
Calcium, ionised [mmol/L]	1.21
Haemoglobin [g/dL]	15.3

Transport was initiated, and monitoring with cNIRS and invasive blood pressure was continued until hospital admission. The patient required push-dose noradrenaline support and was admitted to the intensive care unit with stable hemodynamics.

Coronary angiogram revealed no significant lesions. Initial computed tomography showed no underlying pathology besides myocardial hypertrophy. On day 4, the patient was extubated and neurologically fully intact. For secondary prophylaxis, he received a subcutaneous implantable defibrillator during the hospital stay. Follow-up after 6 months confirmed complete recovery.

## Discussion

This is, to our knowledge, the first paediatric case of DSED. In Germany, the legal age is 18, and patients are treated according to the PLS guidelines if the age is known to be <18.^5^ Although shockable rhythms are less common in this age group when compared to adults, there are underlying conditions like cardiomyopathies that predispose to cardiac arrhythmias. Such cases, though rare, highlight the need for effective treatment strategies when VF proves refractory, including pad position change, as recommended by current adult advanced life support guidelines, and, potentially, DSED as a rescue strategy.^6^

In our case, the use of DSED resulted in sustained ROSC and ultimately, the patient could be stabilised and survived without neurological impairment. This demonstrates the potential benefit of a DSED strategy in paediatric patients with refractory VF.

Current PLS guidelines mention that a vector change (antero-posterior pad placement) could improve outcome and defibrillation success in refractory VF.^5^ A vector change strategy may also have resulted in termination of VF in the case presented, but it was not applied. The team opted for DSED as a second defibrillator is always present at an OHCA in this EMS region, and likely delivered a higher cumulative current to the myocardium.^7^

There are technical challenges associated with delivering DSED. In the DOSE-VF study protocol, DSED was defined as delivering two shocks within 1 s, but not simultaneously. Recent animal and observational data suggest that a short interval between discharge of the two defibrillators (10–100 ms) optimises efficacy of DSED.^4^, ^8^, ^9^ The manufacturer of the defibrillators used in this case has stated that DSED can be used without risk of damaging the defibrillator and suggested switching to non-synchronised mode to enable defibrillation in rapid succession. Using defibrillators in synchronised mode may result in an unpredictable inter-shock interval, as both devices delay shock delivery while awaiting detection of an R wave. In our case, the team did not adhere to this recommendation, highlighting the need for repeated training to optimise defibrillation success when using DSED.

Our patient was treated with a bundle of advanced prehospital interventions considered standard of care in this specialised emergency medical service system, but not commonly used in OHCA yet. The current paediatric life support guidelines recommend ECMO for refractory OHCA, which was on standby in this case.^5^

Notably, the patient’s complete neurological recovery underscores that timely reversal of VF and high-quality post-arrest care can result in good outcomes even after prolonged resuscitation. The effect of DSED on the long-term neurological intact outcome remains hard to isolate, but this case suggests it can be a successful rescue strategy when standard defibrillation fails.

Current research priorities regarding DSED don’t include the paediatric population, and data are scarce.^10^ Because refractory VF is rare in this cohort, conducting single- or multicentre trials will be difficult. To overcome this, registries should be used to capture such cases to facilitate high-quality observational studies.

## Consent

Written informed consent was obtained from the patient and his family for publication of this case report.

## CRediT authorship contribution statement

**F. Weilbacher:** Writing – original draft, Visualization, Project administration, Investigation. **D. Matthé:** Writing – review & editing, Investigation. **J. Fricke:** Writing – review & editing, Investigation. **E. Amr:** Writing – review & editing, Investigation. **M. Dietrich:** Writing – review & editing, Investigation. **E. Popp:** Writing – review & editing, Project administration. **S. Katzenschlager:** Writing – original draft, Visualization, Supervision, Data curation.

## Funding

No funding was received for this publication.

## Declaration of competing interest

SK is the Co-Chair of the ERC PLS SEC and a member of the ILCOR PLS Taskforce.

All other authors declare that they have no competing interests.

## Data Availability

Data are available from the corresponding author on reasonable request.
